# Botulinum toxin A injection into the anterior belly of the digastric muscle increased the posterior width of the maxillary arch in developing rats

**DOI:** 10.1186/s40902-019-0203-7

**Published:** 2019-05-06

**Authors:** Janghoon Ahn, Seong-Gon Kim, Min-Keun Kim, Insan Jang, Hyun Seok

**Affiliations:** 1 Department of Dentistry, College of Medicine, Hallym University, Chuncheon, 24252 South Korea; 2 Department of Oral and Maxillofacial Surgery, College of Dentistry, Gangneung-Wonju National University, Gangneung, 25457 South Korea; 3 Department of Orthodontics, College of Dentistry, Gangneung-Wonju National University, Gangneung, 25457 South Korea; 4 Department of Oral and Maxillofacial Surgery, Chungbuk National University Hospital, Cheongju, 28644 South Korea

**Keywords:** Botulinum toxin A, Anterior belly of digastric muscle, Maxillofacial bone

## Abstract

**Background:**

The purpose of this study was to evaluate the effects of botulinum toxin A (BTX) injection into the anterior belly of the digastric muscle on a growing rat.

**Methods:**

Ten Sprague Dawley rats were used in this study. When the rats were 13 days old, 0.5 units of BTX was injected into the anterior belly of the digastric muscle for the experimental group (*n* = 5). For the control, the same volume of normal saline was injected (*n* = 5). The rats were sacrificed at 60 days old, and the skulls were harvested for micro-computed tomography (μCT) analysis.

**Results:**

In anthropometric analysis, the zygomatic arch and mandibular bi-condylar width were significantly lower in the experimental group than those in the control group (*P* = 0.025 and 0.027, respectively). The maxillary point width was significantly higher in the experimental group than that in the control group (*P* = 0.020).

**Conclusion:**

BTX injection into the anterior belly of the digastric muscle had effects on the maxillofacial bony width in growing rats.

## Background

Botulinum toxin A (BTX) paralyzes the muscle and has been used for therapeutic purposes [1]. In the maxillofacial area, BTX injection is considered a cosmetic therapy and it has been used for the removal of wrinkles or the reduction of muscle volume [2]. Recently, the indication for BTX injection has become much wider [1, 3]. BTX injection into the maxillofacial area has been used for the treatment of orofacial pain [4], as well as for the prevention of open bite [4] or plate fracture [5]. As the dental arch is positioned in the balanced line between the buccinator and tongue muscles, control of muscle power will be beneficial during orthodontic treatment [6].

Orthodontic treatment can be classified as adult orthodontics and child orthodontics. In the case of child orthodontics, the orthodontist should consider individual growth patterns [7] as broken balance among muscles may result in skeletal originated malocclusion. Many kinds of orthopedic appliances have been developed for the correction or prevention of skeletal malocclusion [8]. However, the results of orthopedic treatment have been mostly disappointing [9]. Chin cup is an orthopedic appliance and used for the retraining of mandibular overgrowth [10]—a common complication of chin cup or facemask therapy is mandibular clockwise rotation [9].

There may be various reasons for the failure of an orthopedic appliance, one of which may be a failure in the control of soft tissue growth potential [9]. Compared to the orthopedic appliance, distraction osteogenesis increases not only bone volume but also soft tissue volume [11]. In spite of its effect on growing patients, distraction osteogenesis also has a high rate of postoperative relapse [12]. Soft tissue originated tensional force may induce appositional bone growth [13]. In contrast, reduced tensional force by BTX injection may result in reduced bone growth [14, 15].

The anterior belly of the digastric muscle, as a member of the suprahyoid muscle group, is directly involved in masticatory activity, such as mastication, speech, and swallowing [16, 17]. The major role of the digastric muscle is in the opening of the mouth and depression of the mandible [18]. The mandible is rotated in a clockwise direction during opening of the mouth [18, 19]. In previous studies, the resection of the digastric muscle was shown to induce the notable changes in the mandible position and growth pattern [19]. It reduces the bone size and induces the upward positioning of the mandible [19]. In another study, release of the digastric muscle did not induce a significant change in mandibular length [20].

In a previous study, BTX injection into the masseter and temporalis muscles in growing rats resulted in reduced mandibular growth in the injected side [14, 15]. However, the effect of BTX injection into the anterior belly of the digastric muscle in growing rats has not been studied before. The aim of this study was to examine the effects of BTX injection into the anterior belly of the digastric muscle in growing rats.

## Methods

### Animal experiment and design

This study was approved by the Institutional Animal Care and Use Committee of the Gangneung-Wonju National University, Gangneung, Korea (2016-24). The experimental design and detailed procedure was in accord to our previous publication [15]. Ten Sprague Dawley rats were used in this study and divided into control and experimental groups, with five rats being used in each group. Fifty units of BTX (Botulax® 50, botulinum toxin type A, HUGEL, Chuncheon, Korea) were diluted with 50 ml saline, prepared as 0.5 units per 0.5 ml. When the rats were 13 days old (before puberty), 0.5 unit of BTX was injected into the anterior belly of the digastric muscle of the experimental group (*n* = 5), and 0.5 ml of normal saline (0.9% sodium chloride) was injected into the same muscle in the control group (*n* = 5). All of the rats were injected on the same day and then sacrificed at 60 days old, 47 days after the injection. The skulls were harvested and fixed with formalin solution for micro-computed tomography (μCT) analysis.

### μCT analysis

The μCT analysis was performed at the Ochang Center of the Korea Basic Science Institute (Ochang, Korea). The skull specimen was taken by an animal PET/CT/SPECT system (Inveon™, Siemens, Malvern, PA, USA). The CT scanner was set to 80 kV voltage for the X-ray tube, 500 μA current for the X-ray source, and 210 ms of exposure time. The detector and the X-ray source were rotated through 360° in 360 rotation steps. The number of calibration exposures was 30. The system magnification was performed over 30.7 mm of the axial field of view (FOV) and over 30.7 mm of the transaxial FOV. The scanned images were reconstructed by Inveon Research Workplace Software (Siemens Healthcare). The structure of the skulls was showed in reconstructed three-dimensional images.

### The anthropometric points of maxillofacial bone and measurement of the distance

**Fig. 1:**
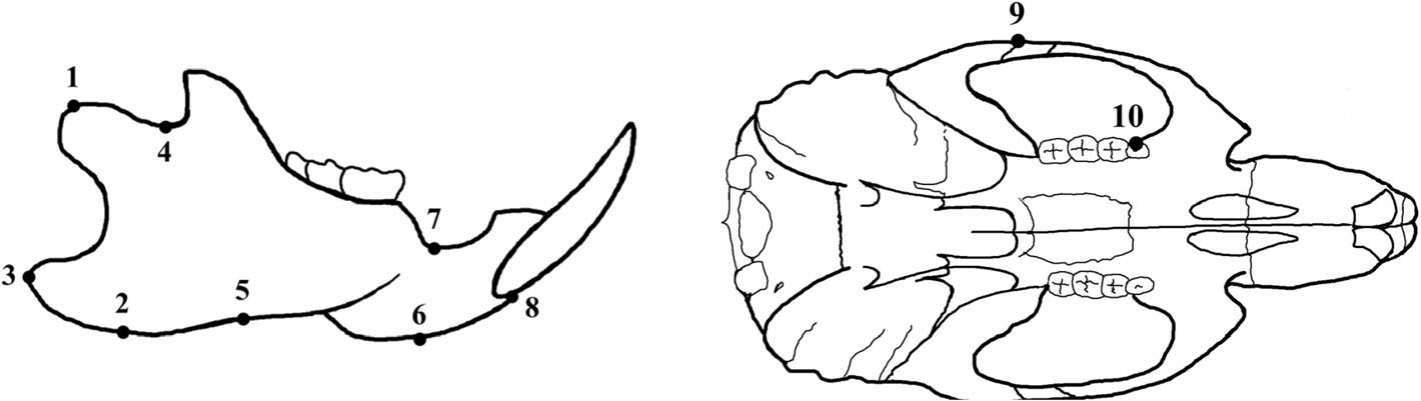
Anthropometric measurement points. (1) Condylion (Cd): most posterior and superior points on the mandibular condyle. (2) Gnathion (Gn): most inferior point of the bony contour of the mandible gonial angle. (3) Gonion (Go): most posterior point of the bony contour of the mandible gonial angle. (4) Coronoid notch (Co): most inferior and concave point of the coronoid notch. (5) Antegonial notch: most superior point of the curvature of the antegonial notch. (6) Menton (Me): most inferior point of the mandibular symphysis. (7) Mandibular alveolar point: most inferior point on the mandibular alveolar crest. (8) Infradentale: most superior and anterior point of the mandibular alveolar bone between the lower central incisors. (9) Zygion (Zy): most external point of the zygomatic arch. (10) Maxillary point (key ridge): most inferior point on the zygomatic process of the maxilla

**Fig. 2:**
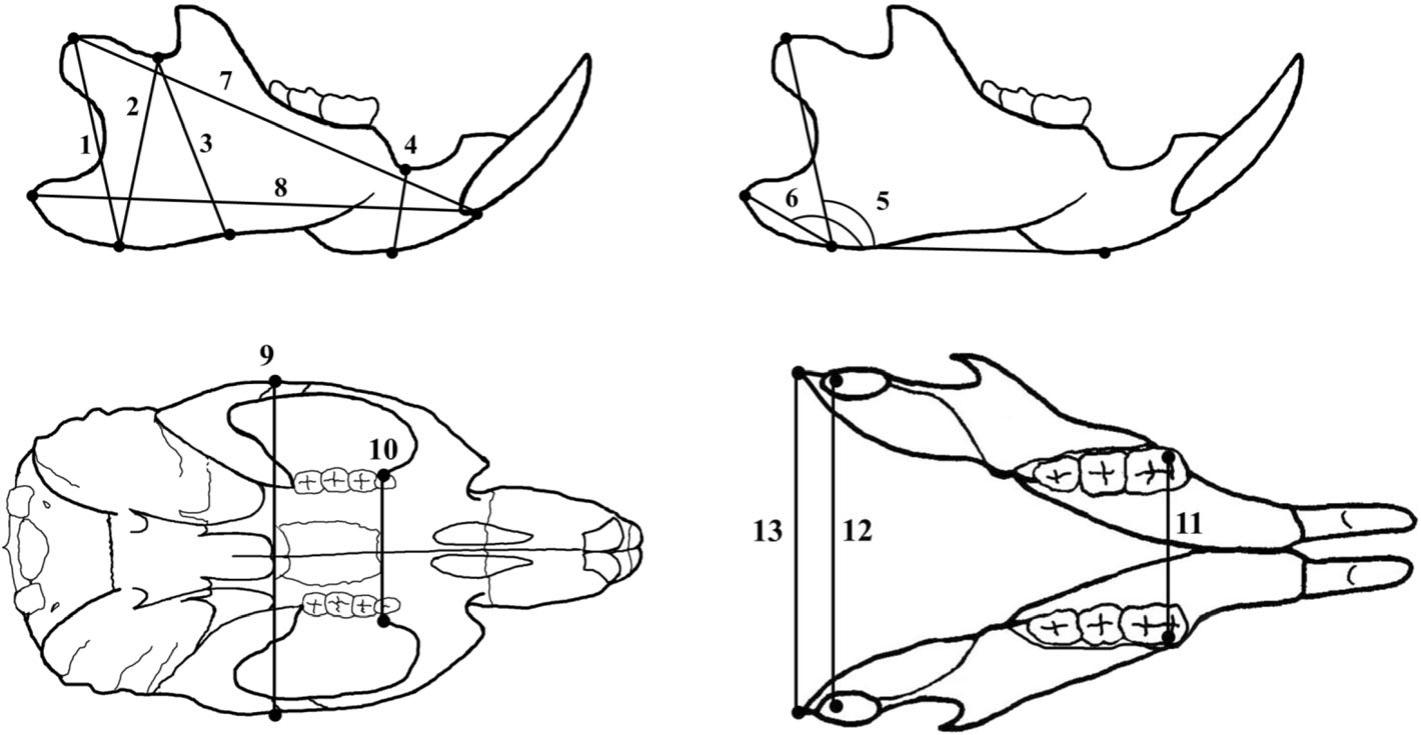
Anthropometric measurements in the maxillofacial bone of rats. (1) Ramus height I: distance between the Cd and Gn. (2) Ramus height II: distance between the Co and Gn. (3) Ramus height III: distance between the Co and antegonial notch. (4) Corpus height: distance between the mandibular alveolar point and Me. (5) Mandibular plane angle I: angle between the line of Cd-Gn and Gn-Me. (6) Mandibular plane angle II: angle between the line of Go-Gn and Gn-Me. (7) Total mandibular length: distance between the Cd and infradentale. (8) Corpus length: distance between the Go and infradentale. (9) Zygomatic arch width: distance between the bilateral Zy. (10) Maxillary point (key ridge) width: distance between the bilateral key ridge point. (11) Mandibular molar width: distance between the bilateral mesiobuccal cusps of the mandibular first molars. (12) Bicondylar width: distance between the bilateral Cd. (13) Bigonial mandibular width: distance between the bilateral Go

The anthropometric points were chosen, and the definition of each point was explained in Fig. 1. The condylion (Cd), gnathion (Gn), gonion (Go), coronoid notch (Co), antegonial notch, menton (Me), mandibular alveolar point, infradentale, zygion (Zy), and maxillary point (key ridge) were pointed, and the distance of each point was measured. The definition of linear distance of each point is explained in Fig. 2. The ramus height I, II, and III; corpus height; mandibular plane angle I and II; total mandibular length; corpus length; zygomatic arch width; maxillary point (key ridge) width; mandibular molar width; bi-condylar width; and bigonial mandibular width were measured.

### Statistical analysis

For the comparison of the variables between the control and experimental groups, an independent samples’ *t* test was used. A *P* value of less than 0.05 was considered statistically significant.

## Results

The anthropometric measurements of the maxillofacial bone in the control and digastric groups were presented in Table 1. There were no significant differences between the groups in the vertical and sagittal anthropometric measurements (*P* > 0.05). In the transverse measurements of the maxillofacial bone, there are significant differences in the zygomatic arch width, maxillary point width, and bi-condylar width between the groups (*P* < 0.05; Table 1). The zygomatic arch width was 12.55 ± 0.21 mm and 12.10 ± 0.30 mm in the control and the experimental groups, respectively (*P* = 0.025). The maxillary point width was 5.04 ± 0.09 mm and 5.95 ± 0.69 mm in the control and the experimental groups, respectively (*P* = 0.020). The bi-condylar width was 10.43 ± 0.14 mm and 10.25 ± 0.07 mm in the control and the experimental groups, respectively (*P* = 0.027). When the measurements between right and left in the same groups were compared, there were no significant differences (*P* > 0.05, data not shown).

**Table 1:** Comparison of the anthropometric measurement of the maxillofacial bone in control and experimental group

Variables	Control	Digastric	*P* value
	Mean ± SD	Mean ± SD	
Vertical measurement (mm)			
Ramus height I	5.44 ± 0.17	5.42 ± 0.18	0.872
Ramus height II	4.09 ± 0.22	3.99 ± 0.20	0.480
Ramus height III	3.87 ± 0.20	3.91 ± 0.16	0.754
Corpus height	2.11 ± 0.33	2.25 ± 0.15	0.283
Sagittal measurement			
Mandibular plane angle I (degree)	107.72 ± 2.20	108.12 ± 2.92	0.791
Mandibular plane angle II (degree)	154.26 ± 4.27	158.17 ± 7.83	0.266
Total mandibular length (mm)	11.50 ± 0.21	11.59 ± 0.30	0.524
Corpus length (mm)	9.91 ± 0.84	10.60 ± 0.38	0.142
Transverse measurement (mm)			
Zygomatic arch width	12.55 ± 0.21	12.10 ± 0.30	0.025*
Maxillary point (key ridge) width	5.04 ± 0.09	5.95 ± 0.69	0.020*
Mandibular molar width	4.46 ± 0.13	4.40 ± 0.22	0.600
Bicondylar width	10.43 ± 0.14	10.25 ± 0.07	0.027*
Bigonial mandibular width	9.76 ± 0.41	9.29 ± 0.26	0.068

(*P < 0.05)

## Discussion

In this study, BTX injection into the anterior belly of the digastric muscle in growing rats showed an increased width of maxillary posterior arch and a decreased width of mandibular condyles (Table 1). Maxillofacial bony growth is affected by the surrounding soft tissue and muscular activity. The hypofunction of the masticatory muscle affects the bone shape and morphology, and it reduces the growth of the maxillofacial bone [21]. BTX as a neurotoxin reversibly induces muscle paralysis and effectively reduces muscle power [22]. When BTX is administered into a masticatory muscle, such as the masseter or temporalis muscle, of growing animals, decreased growth of the maxillofacial bone is observed and the size of bone is significantly reduced [23]. In this study, BTX was injected into the anterior belly of the digastric muscle, and the BTX-injected group showed significant changes in the width of the maxillofacial bone compared to the saline-injected control group (*P* < 0.05, Table 1). To the best of our knowledge, this is the first report on the effects of BTX injection into the anterior belly of the digastric muscle in growing animals.

**Fig. 3:**
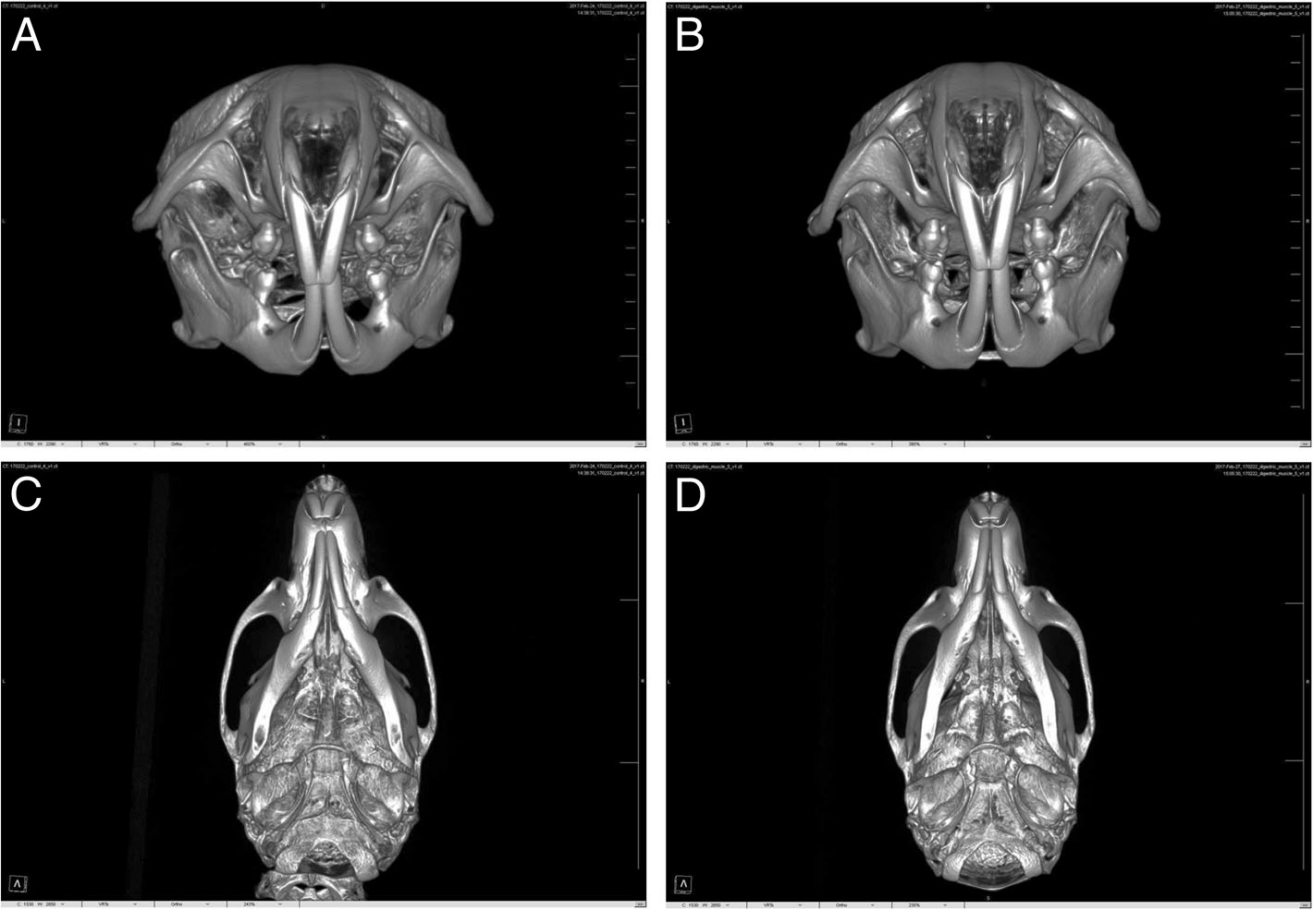
μCT images after the injection of saline for the control group and BTX for the experimental group. Axial view of the control (**a**) and experimental (**b**) groups. Coronal view of the control (**c**) and experimental (**d**) groups

The anterior belly of the digastric muscle, as a member of the suprahyoid muscle group, is directly involved in masticatory activity, such as mastication, speech, and swallowing [16, 17]. The major role of the digastric muscle is in the opening of the mouth and depression of the mandible [18]. The mandible is rotated in a clockwise direction during opening of the mouth [18, 19]. In previous studies, the resection of the digastric muscle was shown to induce the notable changes in the mandible position and growth pattern [19]. It reduces the bone size and induces the upward positioning of the mandible [19]. In another study, release of the digastric muscle did not induce a significant change in mandibular length [20]. In our experiment, the experimental group did not show any significant changes in the mandibular length and height (Table 1). However, there was a significant difference in the width of the maxillofacial bone (Fig. 3). The experimental group showed a significantly decreased zygomatic arch width (*P* = 0.025), increased maxillary point width (*P* = 0.020), and decreased bi-condylar width (*P* = 0.027) compared to the control group.

This result indicates that BTX injections into the digastric muscle in growing rats induced the significant changes in the maxillofacial bony width (Table 1). The cause of the increased bony width in the experimental group can be explained by the change of balance among the masticatory muscles due to the decreased muscle power of the anterior belly of the digastric muscle. The digastric muscle’s role in opening the mouth is shared with other mouth-opening muscles, such as the lateral pterygoid muscle [24]. The jaw-opening movement can be performed by the function of the anterior belly of the digastric muscle and inferior head of the lateral pterygoid muscle [18]. The inferior head of the lateral pterygoid muscle originates from the pterygoid plate of the sphenoid bone and inserted into the neck of the condyle [25]. It induces the sliding movement of the condyle head along the articular eminence and contributes to the condyle rotation and mandible protrusion [26]. The hypofunction of a specific masticatory muscle influences the other masticatory muscle’s power, thus acting synergistically with the weakened muscle. This weakness can lead to the increase of synergistic muscle activity to compensate for the muscle weakness [27]. Based on this fact, the hypofunction of the anterior belly of the digastric muscle could affect the activity of the lateral pterygoid muscle that acts synergistically during jaw opening.

**Fig. 4:**
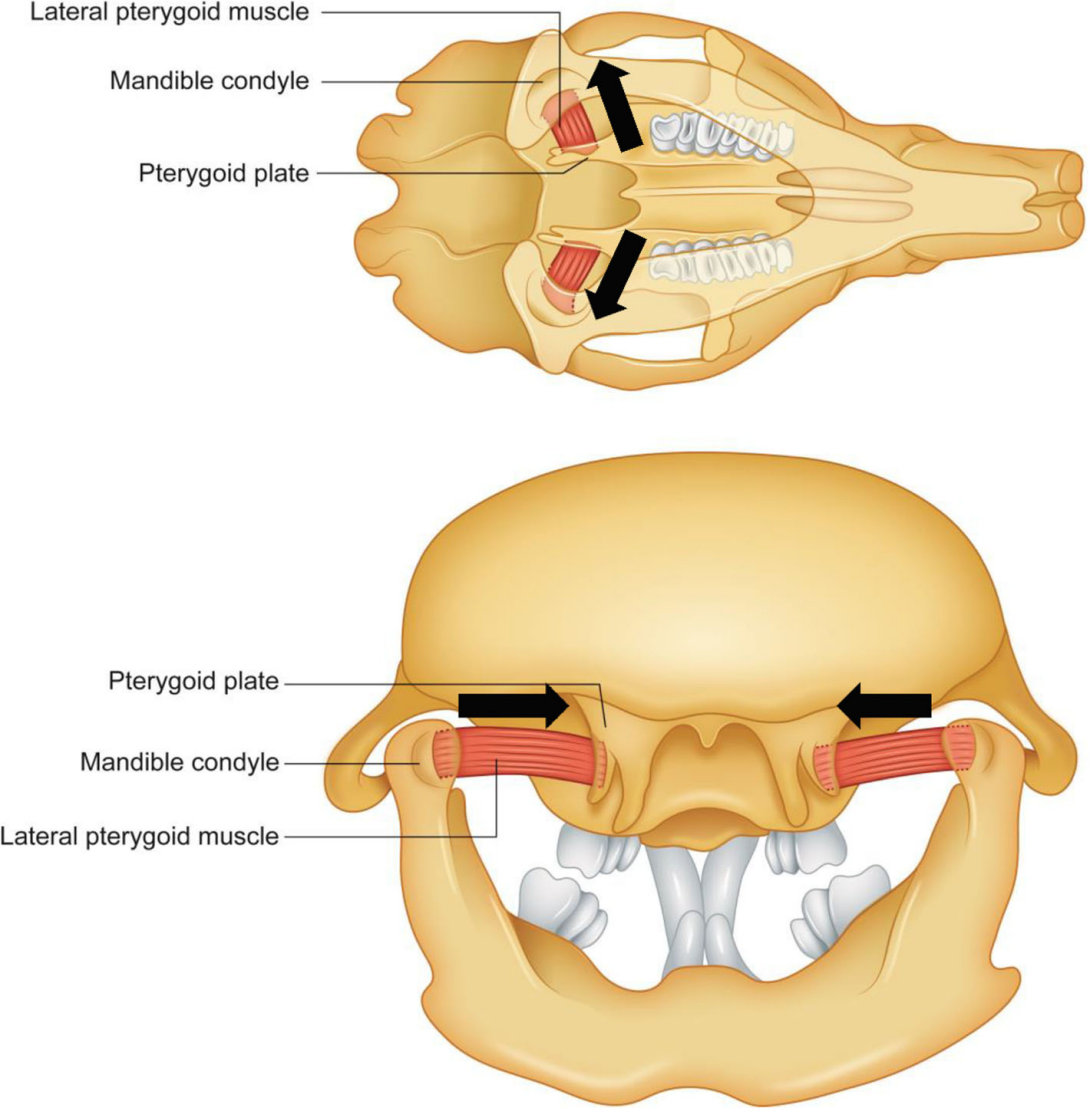
The schematic illustration of the effect of the lateral pterygoid muscle hyperactivity to the growth of the mandibular condyle and maxillary width. The hyperactivity of the lateral pterygoid muscle generates the tensional force to the mandibular condyle and pterygoid plate (black arrow). It contributes to the decrease of the bi-condylar width and increase of the maxillary width according to the posterolateral direction of the force

The distance between the most posterior and superior points of each condyle was significantly decreased in the experimental group (*P* = 0.027). Reduced activity of the digastric muscle could be compensated by elevated activity of the lateral pterygoid muscle. In previous studies, the hyperactivity of the lateral pterygoid muscle was shown to affect the growth and development of the mandibular condyle [28]. Electronic stimulation of the lateral pterygoid muscle increases the muscle activity, and the maturation and calcification, of the chondrocyte in the mandibular condyle [28]. The resection of the lateral pterygoid muscle significantly reduces the growth of condyle cartilage and the mitotic activity of the cartilage [29, 30]. The lateral pterygoid muscle is inserted in the mandibular condyle and disc from the lateral surface of pterygoid plate [25]. The activity of this muscle affects the condyle and disc development. However, the effect on the condyle growth direction has not been adequately studied. Considering the direction of tension force applied to the condyle head during lateral pterygoid muscle action [25], hyperactivity of the lateral pterygoid muscle could change the growth direction of the mandibular condyle, which might lead to inter-condylar width decrease in the experimental group (Fig. 4).

The width of the maxillary point was significantly increased in the experimental group (*P* = 0.020). The maxillary point was defined as the most inferior point on the zygomatic process of the maxilla, and the changes in the maxillary width can be confirmed by the distance of this point [21]. The transverse growth of the maxilla is affected by the maturation of the mid-palatal suture [31]. Before the fusion of this suture, the maxilla width could be changed by applied mechanical force on the palate [32]. The expansive mechanical force using an orthopedic appliance encourages bony remodeling in the mid-palatal suture of rats [33]. Orthodontists have used the palatal extension appliance to correct a narrow maxilla in adolescent patients [34]. If the power of the lateral pterygoid muscle is elevated to compensate for the digastric muscle hypofunction, the pterygoid plate receives the tension force to the posterolateral direction parallel to the muscle direction and it may contribute to the increase of the maxillary width (Fig. 4).

The transverse width of the maxilla and growth of the mid-palatal suture is affected by the masticatory muscle function and occlusal force on the tooth [35]. Rats on a hard food diet show a greater transverse dimension of the maxilla and dental arch due to the increased occlusal loading [36]. The rats that had masticatory hypofunction showed significantly decreased maxillary width compared to the normal rats [21]. The hypofunction of the digastric muscle can affect dental occlusion through changes of the mandibular position [24]. The resection of the suprahyoid muscle induces the upward positioning of the mandible, and the paralysis of the digastric muscle induces the counter-clockwise rotation of the mandible [19, 24]. This positional change of the mandible contributes to establishment of the stable dental occlusion and effective masticatory activity. In clinical practice, BTX injection into the digastric muscle has been used for the treatment of malocclusion [24]. The patient who has an anterior open-bite after mandible fracture surgery receives BTX into the digastric muscle, and the counter-clock wise rotation of the mandible and stable occlusion can be achieved after treatment [1]. The patient who has BTX injected into the digastric muscle after orthognathic surgery shows more stable occlusion and reduced postoperative relapse [1]. According to the previous study, BTX injection into the digastric muscle is an effective treatment for stable occlusion and it will lead to an increase of occlusal loading on the tooth [1, 24]. This improved masticatory activity may contribute to an increase in the transverse width of the maxilla.

In this study, BTX was given subcutaneously by insulin syringe. As the animal was too small, the application of image guiding technique was impossible. BTX was expected to diffuse and influence on the muscles adjacent to injection site. As an isolated effect could not be expected in BTX injection, the application of image guiding technique was of little value. In addition, the suprahyoid muscles do group function. Thus, minute leakage of BTX into other suprahyoid muscles would not change the conclusion of this study.

## Conclusion

In this study, we administrated BTX into the anterior belly of the digastric muscle of growing rats and it induced a significant change in the transverse width of the maxillofacial bone. The changes in the transverse width could possibly be due to the change of the masticatory muscle activity to compensate for the digastric muscle hypofunction. As the joint structure of rats is different from that of humans, BTX injection effects on growing humans may be different from the results of current animal experiments. Additional extensive studies should be followed for clinical applications.

## Abbreviations

BTX: Botulinum toxin A
μCT: Micro-computed tomography
